# Hyponatremia Is Associated With Post-thrombolysis Hemorrhagic Transformation and Poor Clinical Outcome in Ischemic Stroke Patients

**DOI:** 10.3389/fnmol.2022.879863

**Published:** 2022-07-18

**Authors:** Ling He, Zhen-Ni Guo, Yang Qu, Hang Jin

**Affiliations:** ^1^Stroke Center, The First Hospital of Jilin University, Changchun, China; ^2^Department of Neurology, Stroke Center, Clinical Trial and Research Center for Stroke, The First Hospital of Jilin University, Changchun, China

**Keywords:** hyponatremia, hemorrhagic transformation, prognosis, intravenous thrombolysis, ischemic stroke

## Abstract

**Objective:**

Hyponatremia is the most common electrolyte disorder encountered in patients with neurological conditions, such as stroke. Studies have shown that it is associated with worse clinical outcomes and increased mortality in acute ischemic stroke (AIS). However, the role of hyponatremia has not been elucidated in patients with AIS who received intravenous thrombolysis (IVT) therapy. Therefore, this study aimed to investigate the effect of serum sodium levels on the clinical outcome and hemorrhagic transformation (HT) in patients with AIS who received thrombolytic therapy.

**Methods:**

Patients diagnosed with AIS who received IVT therapy between May 2015 and December 2020 were included in this study. All patients were screened for serum sodium levels immediately after hospital admission, before IVT therapy. The occurrence of HT was evaluated using computed tomography (CT) 24 ± 2 h after thrombolysis. Then, 3-month clinical outcomes were obtained by telephone calls or outpatient visits, and poor 3-month clinical outcomes were defined as modified Rankin Scale scores ≥3. The effects of serum sodium levels on the clinical outcome and HT were assessed using the multivariate logistic regression analysis.

**Results:**

Of the 963 included patients, 82 (8.5%) had hyponatremia, 157 (16.3%) developed HT, and 333 (34.6%) had poor 3-month outcomes. Of the 82 patients with hyponatremia, 21 (25.6%) developed HT, and 39 (47.6%) had poor 3-month outcomes. Patients with hyponatremia had a higher incidence of post-thrombolysis HT (25.6 vs. 15.4%, *p* = 0.017) and worse clinical outcome (47.6 vs. 33.4%, *p* = 0.01) than those with normal serum sodium levels. Patients had significantly lower serum sodium levels in those with HT [138.4 (136.4–140.3, IQR) vs. 139.0 (137.2–140.7, IQR) mmol/L, *p* = 0.019] and poor 3 month outcome [139.0 (137.2–140.7) vs. 138.4 (136.7–140.3) mmol/L, *p* = 0.005] than those without. After adjusting for major covariates, the multivariate logistic regression analysis revealed that lower serum sodium levels were independently associated with an increased risk of HT [odds ratio (OR) = 1.804; 95% *CI*: 1.048–3.105] and poor 3-month outcome (*OR* = 1.647; 95% *CI*: 1.012–2.679).

**Conclusion:**

Lower serum sodium level was an independent risk factor for post-thrombolysis HT and poor clinical outcome in patients with AIS who received thrombolytic therapy.

## Introduction

As the annual incidence rate increases, acute ischemic stroke (AIS) is becoming one of the leading causes of mortality and long-term disability in China (Yang et al., 2013). In the early period (≤4.5 h after stroke onset), intravenous thrombolysis (IVT) with the recombinant tissue plasminogen activator (rt-PA) is currently recognized as the best treatment (Powers et al., 2018; Navarro-Oviedo et al., 2019). However, IVT-induced coagulation dysfunction and blood-brain barrier (BBB) disruption may increase the risk of hemorrhagic transformation (HT) and can even lead to symptomatic intracranial hemorrhage (Wang et al., 2015). Thus, it is of critical importance to identify the modifiable risk factors of HT and clinical prognosis to reduce associated death or long-term disability.

Neurological disorders, especially stroke, are frequently complicated by electrolyte imbalances. Discrepancies in serum sodium are the most common electrolyte imbalance in such patients (Saleem et al., 2014; Ehtesham et al., 2019). Incidence of hyponatremia in stroke has been reported to be 11–35% in the literature (Fofi et al., 2012; Mahesar et al., 2019). Accumulating evidence has demonstrated that hyponatremia is an independent risk factor for poor prognosis and mortality in patients with ischemic stroke (Mahesar et al., 2019; Shima et al., 2020). However, very few studies have explored the effect of serum sodium levels on clinical outcomes and the incidence of HT in patients with AIS who received IVT. Therefore, we aimed to explore this effect using our unit prospective database.

## Materials and Methods

### Patients

All patients with AIS who received IVT with rt-PA in the stroke unit of The First Hospital of Jilin University between May 2015 and December 2020 were recruited for this study. The diagnosis of ischemic stroke was made by experienced neurologists and was confirmed using computed tomography (CT) and/or magnetic resonance imaging (MRI; Hurford et al., 2020). Intravenous rt-PA was used within 4.5 h of the symptom onset for all appropriate patients after strict inclusion and exclusion criteria according to the ESO guidelines (Berge et al., 2021). After thrombolysis, all patients received standard medical treatment and general care (Powers et al., 2019). We excluded patients with no available baseline data or no 3-month follow-up information. This study was approved by the Ethics Committee of The First Hospital of Jilin University (2016-294). A written informed consent was obtained from all participants who had the right to withdraw from the study at any point.

### Data Collection

We recorded baseline characteristics, such as demographics, vascular risk factors, previous medication history, clinical features on admission, laboratory tests, and treatment. Cigarette smoking was defined as having smoked at least 1 cigarette per day for 6 months or more (Tong et al., 2016). Alcohol consumption was defined as consuming 15 g or more alcoholic drinks per day in the previous year (Lemarchand et al., 2015). A previous history of drug use was defined as the regular administration of certain drugs before admission. Bridging therapy was defined as mechanical thrombectomy performed after IVT (Wang et al., 2011). Coronary heart disease was defined as a history of coronary heart disease or a clinical diagnosis of coronary heart disease during hospitalization (Wang et al., 2011). Hypertension was defined as a history of hypertension, taking oral antihypertensive drugs, or a clinical diagnosis of hypertension during hospitalization (Wang et al., 2011). Diabetes mellitus was defined as a history of diabetes mellitus, oral hypoglycemic agents/insulin use, or a clinical diagnosis of diabetes mellitus during hospitalization (Wang et al., 2011). A previous history of stroke was defined as a history of transient ischemic attack, ischemic stroke, intracerebral hemorrhage, or subarachnoid hemorrhage (Wang et al., 2011). We screened serum sodium levels immediately after hospital admission for all patients before IVT, and hyponatremia was defined as serum sodium levels <135 mmol/L (Avila, 2014; Mahesar et al., 2019; Potasso et al., 2022). CT and MRI were performed routinely for all patients 24 ± 2 h after thrombolysis, as well as in individual cases of neurological deterioration. HT was determined according to the European-Australasian Acute Stroke Study (ECASS) II classification criteria (Larrue et al., 2001). The 3-month follow-up data were obtained either through outpatient visits or telephone calls with patients and/or relatives by a trained stroke center nurse. We defined modified Rankin Scale (mRS) scores ≥3 as poor outcomes and mRS scores ≤2 as favorable outcomes.

### Statistical Analysis

Normality tests for statistical analysis of all continuous variables were performed using the one-sample Kolmogorov–Smirnov test, and the results are presented as mean with standard deviation (SD) for normally distributed data or median with interquartile range (IQR) for non-normally distributed data. Intergroup differences were compared using unpaired *t*-tests or Mann–Whitney *U*-tests. For categorical variables, the data are expressed as the frequency with percentage, and Pearson’s chi-square test or Fisher’s exact probability test was used for intergroup comparisons. In this analysis, the serum sodium levels were not normally distributed, so Mann–Whitney *U*-tests were used to compare intergroup differences. To explore a potential independent association between serum sodium levels and HT and 3-month outcomes, the measured serum sodium levels were entered into logistic regression models in two formats: as a continuous variable [in which case odds ratio (*OR*) was calculated per 1 mmol/L increase] or as a binary variable. Univariate statistical analyses were performed first, and variates with *p* < 0.1 were included in multivariate logistic regression models. Confidence intervals (*CI*s) were established at 95%, and a two-tailed *p* < 0.05 was considered statistically significant for all statistical tests. Data were analyzed using Statistical Program for Social Sciences version 26.0 (SPSS, IBM, West Grove, PA, United States).

## Results

### Baseline Characteristics

**FIGURE 1:**
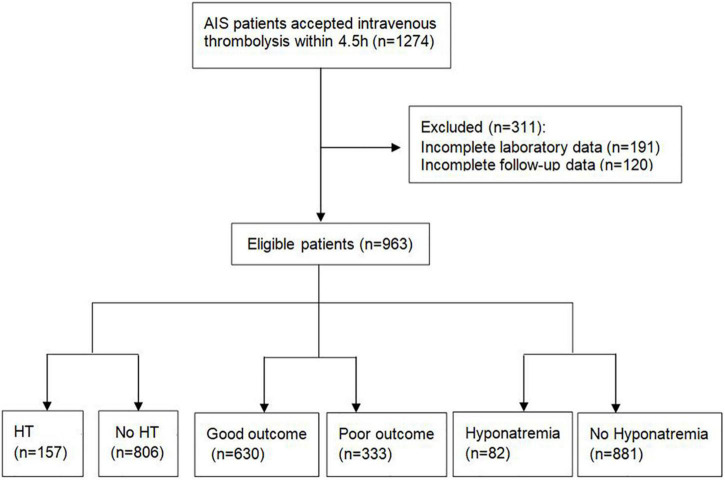
A flowchart of the study. AIS, acute ischemic stroke; HT, hemorrhagic transformation.

A total of 1,274 patients with AIS who received IVT treatment were screened. A total of 120 patients were lost to follow-up and 191 patients had no available baseline data. Finally, 963 patients were included in this study (Figure 1) and their baseline characteristics are shown in Table 1. Of the included participants, 689 (71.5%) were men, and the average age was 61.8 ± 11.8 years. The baseline National Institutes of Health Stroke Scale (NIHSS) score was 8 (5–12, IQR). The mean time from stroke onset to IVT infusion was 180 (141–231, IQR) min. The average serum sodium concentration across all patients was 138.9 (137.0–140.6, IQR) mmol/L. Of the 963 patients, 157 (16.3%) had HT, 333 (34.6%) had poor 3-month outcomes, and 82 (8.5%) had hyponatremia.

**TABLE 1:** Baseline characteristics of the patients with and without hyponatremia.

Variables	Total (*n* = 963)	Hyponatremia (*n* = 82)	Without hyponatremia (*n* = 881)	*P*
**Demographics**				
Age (year)	62 (54–70)	65 (54–72)	62 (54–70)	0.160
Sex [male, n (%)]	689 (71.5)	65 (79.3)	624 (70.8)	0.105
**Vascular risk factors**				
Cigarette smoking, n (%)	523 (54.3)	48 (58.5)	475 (53.9)	0.541
Alcohol consumption, n (%)	442 (45.9)	35 (42.7)	407 (46.2)	0.422
Coronary heart disease, n (%)	169 (17.5)	16 (19.5)	153 (17.4)	0.625
Hypertension, n (%)	484 (50.3)	39 (47.6)	445 (50.5)	0.596
Diabetes mellitus, n (%)	178 (18.5)	21 (25.6)	157 (17.8)	0.082
Previous ischemic stroke, n (%)	114 (11.8)	7 (8.5)	107 (12.1)	0.333
Atrial fibrillation, n (%)	73 (7.6)	6 (7.3)	67 (7.6)	0.865
Body mass index (BMI)	24.2 (23.1–25.4)	24.36 (22.8–25.9)	24.22 (23.1–25.4)	0.746
**Previous medication**				
Antihypertensive medication, n (%)	364 (37.8)	30 (36.6)	334 (37.9)	0.813
Hypoglycemic drugs/insulin, n (%)	159 (17.5)	20 (24.4)	139 (15.8)	0.045
Antithrombotic agents, n (%)	118 (12.3)	13 (15.9)	105 (11.9)	0.299
Anticoagulant agents, n (%)	9 (0.9)	1 (1.2)	8 (0.9)	0.779
**Clinical features on admission**				
Admission NIHSS score	8 (5–12)	9 (6–13)	8 (5–12)	0.022
Admission SBP (mmHg)	156 (140–175)	161 (137–179)	156 (140–175)	0.927
Admission DBP (mmHg)	90 (79–100)	84 (75–98)	90 (80–100)	0.02
DNT (minutes)	180 (141–231)	195 (154–250)	180 (140–230)	0.048
**Laboratory tests**				
Homocysteine (μmol/L)	14.2 (11.0–21.1)	16.8 (11.5–31.2)	14.0 (10.9–20.3)	0.016
Serum sodium (mmol/L)	138.9 (137.0–140.6)	133.5 (132.9–134.3)	139.1 (137.5–140.8)	<0.001
LDL-C (mmol/L)	2.9 (2.4–3.4)	2.82 (2.17–3.44)	2.95 (2.43–3.43)	0.346
Blood glucose (mmol/L)	7.4 (6.4–9.1)	8.0 (6.6–11.7)	7.4 (6.4–9.0)	0.012
**Treatment**				
Bridging therapy	77 (8.0)	9 (11)	68 (7.7)	0.298
0.6 mg/kg rt-PA	214 (22.2)	18 (22)	196 (22.2)	0.918
**Outcome**				
HT	157 (16.3)	21 (25.6)	136 (15.4)	0.017
Poor 3-month outcome	333 (34.6)	39 (47.6)	294 (33.4)	0.01

*Data are expressed as mean ± standard deviation (SD)/median and IQR or n (%). HT, hemorrhagic transformation; NIHSS, National Institutes of Health Stroke Scale; SBP, systolic blood pressure; DBP, diastolic blood pressure; LDL-C, low density lipoprotein cholesterol; DNT, door to needle time; rt-PA, recombinant tissue plasminogen activator.*

### Characteristics Between Patients With and Without Hyponatremia

**FIGURE 2:**
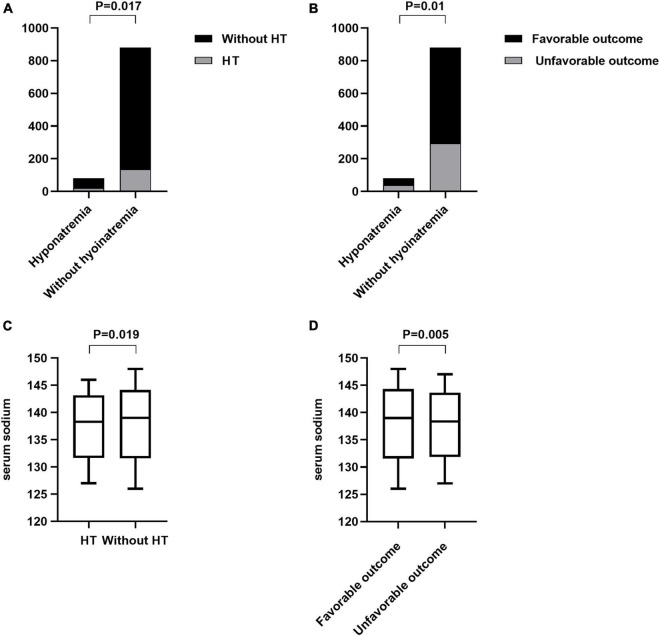
Comparison of the endpoints between patients with and without hyponatremia and the comparison of serum sodium levels between different endpoint groups. **(A)** Incidence of HT between hyponatremia and without hyponatremia groups (25.6% vs. 15.4%, *p* = 0.017). **(B)** Incidence of the poor 3-month outcome between patients with and without hyponatremia (46.7% vs. 33.4%, *p* = 0.01). **(C)** Comparison of the serum sodium levels between the HT and without HT groups {138.4 [136.4–140.3, interquartile range (IQR)] vs. 139.0 (137.2–140.7, IQR) mmol/L, *p* = 0.019}. **(D)** Comparison of the serum sodium levels between the different prognosis groups [139.0 (137.2–140.7) vs. 138.4 (136.7–140.3) mmol/L, *p* = 0.005].

Patient characteristics of the hyponatremia and non-hyponatremia groups are compared in Table 1. There was a statistically significant difference in the serum sodium levels between the two groups [133.5 (132.9–134.3, IQR) vs. 139.1 (137.5–140.8, IQR), *p* < 0.001, Table 1]. Hyponatremia patients were usually younger and consisted more of men than normal serum sodium patients. In addition, the hyponatremia group had a higher admission diastolic pressure, higher blood glucose, and higher homocysteine levels than the normal serum sodium group. Furthermore, patients with hyponatremia had higher NIHSS scores on admission and longer door-to-needle times than those without. Twenty-one patients developed HT in the hyponatremia group. We found that patients with hyponatremia were more likely to develop HT than those without hyponatremia (25.6 vs. 15.4%, *p* = 0.017, Figure 2A). Thirty-nine participants in the hyponatremia group had poor 3-month outcomes. This rate was significantly higher than that in the normal serum sodium group (47.6 vs. 33.4%, *p* = 0.01, Figure 2B).

### Relationship Between Hyponatremia and Post-thrombolysis Hemorrhagic Transformation

**FIGURE 3:**
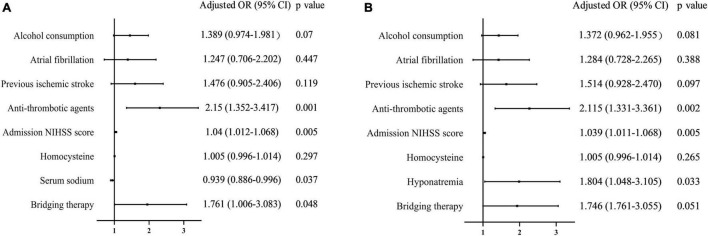
An association between serum sodium levels/hyponatremia and post-thrombolysis HT. **(A)** Association between serum sodium levels and post-thrombolysis HT. **(B)** Association between hyponatremia and post-thrombolysis HT.

Serum sodium levels in patients who developed HT were significantly lower than those in patients who did not [138.4 (136.4–140.3, IQR) vs. 139.0 (137.2–140.7, IQR) mmol/L, *p* = 0.019, Figure 2C]. A univariate analysis showed that HT was associated with alcohol consumption, atrial fibrillation, antihypertensive medication, antithrombotic agents, admission NIHSS score, and serum sodium levels (Table 2). After adjusting for potential confounding factors, the multivariate logistic regression demonstrated that lower serum sodium levels had an independent negative relationship with HT risk (*OR* per 1-mmol/L increase 0.939, 95% *CI* 0.886–0.996, *p* = 0.0037; Figure 3A). Patients with hyponatremia showed a significant increase in risk (*OR* 1.804, 95% *CI* 1.048–3.105, *p* = 0.033, Figure 3B).

**TABLE 2:** Univariate analysis of the baseline factors associated with HT and 3-month outcomes.

Variables	HT			Outcome		
	Yes	No	*p*	Favorable	Unfavorable	*P*
**Demographics**						
Age (year)	63 (54–70.5)	62 (54–70)	0.829	61 (53–69)	65 (56–72)	<0.001
Sex [male, n (%)]	120 (76.4)	569 (70.6)	0.139	449 (71.3)	240 (72.1)	0.793
**Vascular risk factors**						
Cigarette smoking, n (%)	86 (54.8)	437 (54.2)	0.898	344 (54.6)	179 (53.8)	0.801
Alcohol consumption, n (%)	82 (52.2)	360 (47.7)	0.083	291 (46.2)	151 (53.8)	0.802
Coronary heart disease, n (%)	29 (18.5)	140 (17.4)	0.74	108 (17.1)	61 (18.3)	0.648
Hypertension, n (%)	87 (55.4)	397 (49.3)	0.167	302 (47.9)	182 (54.7)	0.045
Diabetes mellitus, n (%)	32 (20.4)	146 (18.1)	0.503	111 (17.6)	67 (20.1)	0.849
Previous ischemic stroke, n (%)	27 (17.2)	87 (10.8)	0.024	63 (10.0)	51 (15.3)	0.016
Atrial fibrillation, n (%)	18 (11.5)	55 (6.8)	0.047	44 (7.0)	4 (8.7%)	0.337
Body mass index (BMI)	24.5 (23.4–25.6)	24.2 (23.0–25.4)	0.473	24.3 (22.9–25.7)	24.2 (23.1–25.2)	0.496
**Previous medication**						
Antihypertensive medication, n (%)	69 (43.9)	285 (36.6)	0.083	222 (35.2)	142 (42.6)	0.024
Hypoglycemic drugs/insulin, n (%)	32 (20.4)	127 (15.8)	0.155	100 (15.9)	59 (17.7)	0.464
Antithrombotic agents, n (%)	34 (21.7)	84 (10.4)	<0.001	71 (11.3)	47 (14.1)	0.201
Anticoagulant agents, n (%)	2 (1.3)	7 (0.9)	0.631	6 (1.0)	3 (0.9)	0.937
**Clinical features on admission**						
Admission NIHSS score	10 (7–14)	8 (5–12)	<0.001	7 (4–10)	11 (7.5–15)	<0.001
Admission SBP (mmHg)	160 (140–177)	156 (139–174)	0.995	156 (140–175)	156 (139–175)	0.279
Admission DBP (mmHg)	93 (82–103)	90 (79–100)	0.105	89 (79–99)	91 (79–100)	0.687
DNT (minutes)	180 (158–232)	180 (139–230)	0.227	180 (142–230)	180 (236–232)	0.899
**Laboratory tests**						
Homocysteine (μmol/L)	15.6 (11.1–23.2)	14.1 (11.0–20.1)	0.098	13.7 (10.8–19.6)	15.6 (11.4–24.5)	0.008
**Serum sodium (mmol/L)**	138.4 (136.3–140.3)	139.0 (137.2–140.7)	0.025	139.0 (137.2–140.7)	138.4 (136.7–140.3)	0.002
**Hyponatremia, n (%)**	21 (13.4)	61 (7.6)	0.019	43 (6.8)	39 (11.7)	0.011
LDL-C (mmol/L)	2.85 (23.4–25.6)	2.96 (2.43–3.43)	0.161	2.9 (2.4–3.4)	2.9 (2.4–3.4)	0.791
Blood glucose (mmol/L)	7.5 (6.7–10.3)	7.4 (6.4–9.0)	0.693	7.3 (6.3–9.2)	7.7 (6.7–9.0)	0.956
**Treatment**						
Bridging therapy	57 (7.1)	449 (71.3)	0.018	42 (6.7)	35 (10.5)	0.038
0.6 mg/kg rt-PA	44 (21)	181 (22.5)	0.667	131 (20.8)	83 (24.9)	0.194
**Outcome**						
HT	157	0		88 (14.0)	69 (20.7)	
Poor 3-month outcome	69 (43.9)	264 (32.8)		630	333	

*Data are expressed as mean ± SD/median and IQR or n (%). HT, hemorrhagic transformation; NIHSS, National Institutes of Health Stroke Scale; SBP, systolic blood pressure; DBP, diastolic blood pressure; LDL-C, low density lipoprotein cholesterol; DNT, door to needle time; rt-PA, recombinant tissue plasminogen activator.*

### Relationship Between Hyponatremia and 3-Month Outcome

**FIGURE 4:**
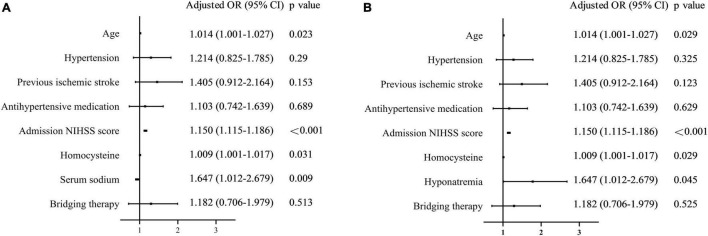
An association between serum sodium levels/hyponatremia and 3-month outcomes. **(A)** Association between serum sodium levels and 3-month outcomes. **(B)** Association between hyponatremia and 3-month outcomes.

Serum sodium levels were lower in the poor 3-month outcome group than in the favorable outcome group [139.0 (137.2–140.7) vs. 138.4 (136.7–140.3) mmol/L, *p* = 0.005, Figure 2D]. A univariate analysis showed that poor 3-month outcomes were associated with age, hypertension, previous ischemic stroke, antihypertensive medication, admission NIHSS score, homocysteine level, and serum sodium level (Table 2). After adjustment, the multivariate logistic regression showed that lower serum sodium levels were independently associated with poor 3-month outcomes (*OR* per 1-mmol/L increase 0.938, 95% *CI* 0.894–0.984, *p* = 0.009; Figure 4A). Patients with hyponatremia had an increased risk of poor 3-month outcomes (*OR* 1.647, 95% *CI* 1.012–2.679, *p* = 0.045, Figure 4B).

## Discussion

In this retrospective study, we evaluated the effect of serum sodium levels on HT and clinical outcomes in 963 patients with AIS who underwent IVT. We found that hyponatremia before IVT was an independent risk factor for post-thrombolytic HT and poor clinical outcomes. Lower serum sodium levels on hospital admission are associated with an increased risk of HT and worse clinical outcomes.

Hyponatremia is a common electrolyte disorder in patients with acute stroke. As previously reported, it occurs in 3.9–45.3% of patients on admission and in 40–45% of patients during hospitalization (Liamis et al., 2019). Stroke-related hyponatremia can be caused by adrenal insufficiency due to pituitary ischemia or hemorrhage, syndrome of inappropriate antidiuretic hormone secretion (SIAD), and cerebral salt wasting (CSW) (Liamis et al., 2019). Cerebro-cardiac syndrome (Moretti et al., 1976) after AIS can also cause hypovolemic hyponatremia. Hyponatremia is frequently a marker of underlying diseases and has long been described as a risk factor for poor prognosis and higher mortality in stroke and cardiovascular diseases (Rodrigues et al., 2014). Wannamethee et al. (1994) published a prospective study of 7,690 middle-aged men who were followed-up over a 9.5-year period and found that serum sodium concentration is a risk factor for stroke. In addition, hyponatremia in the acute stroke stage is reportedly a predictor of 3-year mortality in the first-ever ischemic stroke, independent of other adverse prognostic predictors (Huang et al., 2012). Moreover, Rodrigues et al. (2014) first demonstrated in 2014 that hyponatremia is associated with not only acute mortality but also 3-month and 1-year mortality after stroke. A meta-analysis identified 12 studies with 21,973 patients in 2019 and confirmed that hyponatremia has a significant prognostic value for short-term (within 90 days) and long-term (more than 1 year) prognosis in patients with stroke (Chen et al., 2019). However, the association between serum sodium concentration and clinical outcomes in patients with stroke who receive IVT remains unclear. We found that 8.5% of the participants on admission had hyponatremia, which is comparable with the incidence observed in previous studies (Barkas et al., 2019). As expected, we found that a lower serum sodium concentration had an independent negative relationship with the 3-month outcome in patients with AIS who received IVT, which was consistent with previous studies. Simultaneously, we first found that lower serum sodium concentrations were independently associated with an increased risk of HT in patients with AIS after IVT.

The mechanisms underlying hyponatremia and the clinical outcome of stroke remain unclear. One possible mechanism is that hyponatremia in patients with stroke may exacerbate osmotic brain edema through fluid shifts (Chen et al., 2019). Under physiological conditions, brain osmolality is in equilibrium with the osmolality of extracellular fluids (Giuliani and Peri, 2014). When hyponatremia occurs, the increased ionic gradient between the vascular compartment and interstitial fluid provides the driving force for subsequent ionic edema (Khanna et al., 2014). Furthermore, sodium, as a quantifiable metric of ischemic lesions and tissue recovery, is directly linked to the disrupted Na^+^/K^+^-ATPase activity, which can cause cytotoxic edema (Yin et al., 2020; Helsper et al., 2022). Additionally, studies (Chen et al., 2021) have proved that, under ischemic stroke conditions, BBB dysfunction can result in increased paracellular permeability, directly contributing to the extravasation of blood components into the brain and causing cerebral vasogenic edema. Unfortunately, hyponatremia can accelerate this process *via* a cascade of disrupted ionic homeostasis. It has also been reported that relatively small changes in the brain water content can reflect large changes in the absolute water content of the brain and brain swelling (Keep et al., 2012). Thus, cerebral edema caused by hyponatremia can result in massive cerebral swelling, subsequently increased intracranial pressure, rapid neurological deterioration, and increased morbidity and mortality following ischemic stroke (Chen et al., 2021; Guo et al., 2021). Our study found that patients with hyponatremia had higher NIHSS scores at admission than those without hyponatremia. All patients included in our study arrived at the hospital within 4.5 h of the stroke onset. Therefore, in the early stages of stroke, hyponatremia may quickly affect osmotic brain edema, aggravate cellular edema and vasogenic edema, and further aggravate preexisting neurological deficits. This leads to worse clinical outcomes. The mechanisms of hyponatremia and post-thrombolysis HT remain unknown. Hyponatremia is observed more frequently in patients with hemorrhagic stroke than in those with ischemic stroke. Hyponatremia was found in 18.4% of the patients with ischemic stroke and 25.8% of the patients with hemorrhagic stroke, irrespective of age and sex (Yaghi et al., 2017). HT, especially symptomatic intracerebral hemorrhage, is the most serious complication of IVT (Bosche et al., 2020). Although the exact mechanisms of HT are unknown, previous studies have demonstrated that HT after thrombolysis may be associated mainly with the disruption of the BBB and rt-PA-induced reperfusion injury (Krueger et al., 2015; Rocha et al., 2019). BBB is a key mediator of cerebral homeostasis. BBB disruption plays an important role in vasogenic edema, cell death, neuroinflammation, and other features of ischemic cerebral stroke (Chen et al., 2020; Yang et al., 2021). It has been reported that BBB disruption can be cumulatively deteriorated by ischemia and rt-PA treatment (Liu et al., 2016). In our study, we found that hyponatremia was independently associated with increased risks of post-thrombolysis HT. It has been reported that, in hyponatremia conditions of the process of solute and water transfers, volume perturbations cause cytoskeletal rearrangements, which can break down the tight junctions and endothelial cell integrity (Khanna et al., 2014). Additionally, in the late stage of ionic edema, when vasogenic edema happens, capillaries become fenestrated, tight junctions are disrupted, and reverse pinocytosis occurs to increase the transport of macromolecules into the brain parenchyma, thereby causing the disruption of the BBB, solute influx, more edema, and ultimately, HT (Khanna et al., 2014). However, the exact causes of worse clinical outcomes and the increased incidence of HT observed in patients with hyponatremia who received IVT remain to be determined and require further investigation.

Since an independent association was observed among poor clinical outcomes, post-thrombolysis HT, and hyponatremia, would timely and appropriate management of hyponatremia improve the prognosis of patients with AIS after IVT? Previous studies reported that the serum sodium concentration and stroke mortality have a “U”-typed relation (Wannamethee et al., 1994). Both hyponatremia and hypernatremia have been associated with an early neurological deterioration following stroke (Fofi et al., 2012). However, this was not verified in our study. Nevertheless, our findings imply that hyponatremia is related to the poor 3-month prognosis and an increased risk of HT in patients with AIS who received IVT. More attention should be given to hyponatremia before thrombolytic treatment. Whether sodium supplementation can improve prognosis requires further investigation.

This study had some limitations. The main limitation was the lack of a hypernatremia group because only a few patients had serum sodium concentrations beyond the upper normal limit. Therefore, the effect of hypernatremia in patients with thrombolysis cannot be fully evaluated. Moreover, this was a single-center, retrospective, observational study. Additionally, we could not evaluate and correct for potentially confounding neuroimaging metrics, such as ischemic core volume before thrombolysis. Finally, we did not explore the relationship between sodium level and secondary intracerebral hemorrhage in a few samples.

## Conclusion

In conclusion, to the best of our knowledge, this is the first study to demonstrate that a lower serum sodium level before IVT is an independent predictor of post-thrombolysis HT and poor clinical prognosis in patients with AIS who underwent IVT. Further randomized controlled trials are needed to determine whether early initiation of sodium supplementation therapy in thrombolytic patients with low serum sodium levels can reduce the risk of HT and can improve prognosis.

## Data Availability Statement

The raw data supporting the conclusions of this article will be made available by the authors, without undue reservation.

## Ethics Statement

The studies involving human participants were reviewed and approved by the Ethics Committee of The First Hospital of Jilin University (2016-294). The patients/participants provided their written informed consent to participate in this study.

## Author Contributions

HJ and Z-NG conceived the perspective of the work. LH and YQ collected and analyzed the data. LH searched the literature and drafted the manuscript. YQ and Z-NG critically revised the article. HJ was responsible for checking the whole manuscript. All authors contributed to the article and approved the submitted version.

## Conflict of Interest

The authors declare that the research was conducted in the absence of any commercial or financial relationships that could be construed as a potential conflict of interest.

## Publisher’s Note

All claims expressed in this article are solely those of the authors and do not necessarily represent those of their affiliated organizations, or those of the publisher, the editors and the reviewers. Any product that may be evaluated in this article, or claim that may be made by its manufacturer, is not guaranteed or endorsed by the publisher.
